# Supplementary material for: Eye features and retinal photoreceptors of the nocturnal aardvark (Orycteropus afer, Tubulidentata)
Source: PLoS One. 2025 Mar 24;20(3):e0314252. doi: 10.1371/journal.pone.0314252 (PMC11932471; doi:10.1371/journal.pone.0314252)
Supplement: S1 Table — (DOCX) [file pone.0314252.s004.docx]

**S1 Table. Aardvark photoreceptor (rod) densities**

| **Animal, counting field position** | **Photoreceptor density** (1/mm²) | **Number of ONL tiers** |
| --- | --- | --- |
|  |  |  |
| **Aardvark 1** |  |  |
| near streak | 207,000 | 8-9 |
| near streak | 200,000 | 8 |
| midperiphery | 186,000 | 9 |
| midperiphery | 203,000 | 8 |
| midperiphery | 189,000 | 8 |
| midperiphery | 214,000 | 8-9 |
| midperiphery | 160,000 | 7 |
| midperiphery | 175,000 | 7 |
| midperiphery | 154,000 | 6 |
| midperiphery | 144,000 | 6-7 |
| periphery | 144,000 | 6 |
| periphery | 137,000 | 6 |
| periphery | 135,000 | 6 |
| periphery | 140,000 | 6-7 |
| periphery | 124,000 | 7 |
| far periphery | 129,000 | 6 |
|  |  |  |
| **Aardvark 2** |  |  |
| near OD | 215,000 | 6-7 |
| near OD | 236,000 | 7 |
| near OD | 238,000 | 6-9 |
| near OD | 245,000 | 7-9 |
| near OD | 182,000 | 5-7 |
| midperiphery | 187,000 | 5-7 |
| midperiphery | 198,000 | 6-7 |
| periphery | 163,000 | 5 |

Photoreceptor nuclei in the outer nuclear layer (ONL) were counted in DAPI-labelled transverse sections. Data for aardvark 1 are from cryostat sections running from the optic disc (OD) to the dorsal retinal periphery, crossing the streak (see S1 Fig). Data for aardvark 2 are from paraffin sections of the whole eye. These sections were central and included the OD, but the orientation (dorsal/ventral or temporal/nasal) was unknown. Densities are rounded to full 1000/mm². As aardvark cone densities are very low, the photoreceptor densities can be equaled to rod densities.
