# Supplementary material for: Eye features and retinal photoreceptors of the nocturnal aardvark (Orycteropus afer, Tubulidentata)
Source: PLoS One. 2025 Mar 24;20(3):e0314252. doi: 10.1371/journal.pone.0314252 (PMC11932471; doi:10.1371/journal.pone.0314252)
Supplement: S2 Table — (DOCX) [file pone.0314252.s005.docx]

**S2 Table. Cone densities in various retinal regions of aardvark 1**

| **Counting region, counting field** | **Cone density**  (1/mm²) | | **%age of dual pigm. cones** | **%age of pure S cones** | **%age of pure L cones** |
| --- | --- | --- | --- | --- | --- |
| Region 1 |  | |  |  |  |
| Data given in Fig. 8 | |  |  |  |  |
| Region 2 |  | |  |  |  |
| Field 2a | 610 | | 91 | 7 | 2 |
| Field 2b | 630 | | 94 | 6 | 0 |
| Region 3 |  | |  |  |  |
| Field 3a | 530 | | 96 | 2 | 2 |
| Field 3b | 550 | | 92 | 4 | 4 |
| Field 3c | 550 | | 87 | 6 | 7 |
| Region 4 |  | |  |  |  |
| Field 4a | 610 | | 84 | 13 | 3 |
| Field 4b | 650 | | 81 | 17 | 2 |
| Field 4c | 600 | | 89 | 8 | 3 |
| Region 5 |  | |  |  |  |
| Field 5a | 542 | | 91 | 2 | 7 |
| Field 5b | 603 | | 93 | 4 | 3 |
| Field 5c | 567 | | 98 | 1 | 1 |
| Field 5d | 595 | | 90 | 4 | 6 |
| Field 5e | 534 | | 89 | 3 | 8 |
| Region 6 |  | |  |  |  |
| Field 6a | 840 | | 95 | 5 | 0 |
| Field 6b | 690 | | 88 | 12 | 0 |
| Field 6c | 570 | | 91 | 9 | 0 |
| Region 7 |  | |  |  |  |
| Field 7a | 630 | | 94 | 6 | 0 |
| Field 7b | 680 | | 94 | 2 | 4 |
| Region 8 |  | |  |  |  |
| Field 8a | 950 | | 35 | 65 | 0 |
| Field 8b | 950 | | 36 | 63 | 1 |
| Field 8c | 871 | | 36 | 58 | 6 |
| Region 9 |  | |  |  |  |
| Field 9a (D) | 696 | | ? | ? | 0 |
| Field 9b (D) | 860 | | ? | ? | 1 |
| Field 9c (D) | 708 | | ? | ? | 1 |
| Field 9d (D) | 640 | | ? | ? | 1 |
| Field 9e (D) | 868 | | ? | ? | 0 |
| Field 9f (streak) | 900 | | ? | ? | 1 |
| Field 9g (streak) | 1072 | | ? | ? | 0 |
| Field 9h (streak) | 968 | | ? | ? | 0 |
| Field 9i (V) | 940 | | ? | ? | 0 |
| Field 9j (V) | 844 | | ? | ? | 0 |
| Field 9k (V) | 840 | | ? | ? | 0 |
| Field 9l (V) | 692 | | ? | ? | 0 |
| Field 9m (V) | 612 | | ? | ? | 1 |
| Field 9n (V) | 592 | | ? | ? | 0 |
| Field 9o (V) | 604 | | ? | ? | 0 |

Cone densities and the proportions of dual pigment cones, pure S cones and pure L cones were counted in several counting fields within 9 regions of the right retina of aardvark 1. For the positions of the counting regions, see the schematic drawing S1 Fig. Counting field sizes ranged from 317 µm x 317 µm to 636 µm x 636 µm. In the large temporal region 9, the approximate position of the counting fields is given as near the streak, dorsal of it (D), or ventral of it (V). In region 9, for technical reasons the L opsin label was faint, hence the proportions of dual pigment cones and thus also pure S cones could not be reliably determined and are not given. As pure L cones could be identified due to their higher L opsin content, the total number of cones (i.e., S opsin-expressing cones and pure L cones) could be determined.
