# Supplementary material for: Eye features and retinal photoreceptors of the nocturnal aardvark (Orycteropus afer, Tubulidentata)
Source: PLoS One. 2025 Mar 24;20(3):e0314252. doi: 10.1371/journal.pone.0314252 (PMC11932471; doi:10.1371/journal.pone.0314252)
Supplement: Attachment — Submitted filename: Response to Reviewers.docx [file pone.0314252.s007.docx]

PONE-D-24-50728

Eye features and retinal photoreceptors of the nocturnal aardvark (*Orycteropus afer*, Tubulidentata)

**Response to Reviewers**

Dear Editor, dear Reviewers,

We are very pleased by the reviewers’ positive response to our manuscript, and we greatly appreciate their thoughtful and detailed suggestions for improvement and clarifications. Here are our responses to the specific points raised by the reviewers. The line numbers refer to the marked-up version of the revised manuscript.

**Reviewer #1:**

This manuscript by Peichl L, et al. investigates the anatomical features of the nocturnal aardvark eye and its photoreceptor composition. The authors took advantage of a rare opportunity to obtain aardvark eye samples from a local zoo. Using standard modern techniques, they re-evaluated the findings made over a century ago, corrected previous misconceptions, and provided new descriptions. These include details on the structure of the tapetum lucidum, RPE pigmentation, rod density, rod nuclear structures, cone density, and cone pigment expression. This careful examination of these features provides valuable information about the species-specific adaptations of the eye in this ‘living fossil’ species, contributing to advancing our understanding of the evolutionary origins of the mammalian eye. The manuscript is well-written at a high standard, and the data are presented clearly, with a few exceptions noted in the minor comments below.

Thank you for this positive overall appraisal.

Minor comments
1. (Lines 443-445) ‘The observed range was about 124,000-214,000 photoreceptors (rods)/mm^2^, with densities decreasing from central to peripheral retina.’
Please provide the actual data showing the retinal locations and corresponding rod densities. This information is helpful in assessing how sharply or gradually rod density changes with retinal eccentricity.

We have added Table S1 with the photoreceptor density counts and modified the main text accordingly. Densities were assessed along retinal transverse sections running from near the optic disc to the dorsal periphery (thick black line in the new Fig. S1). The information on retinal localization is limited (near streak, midperiphery, etc.), because the positions of counting fields along the sections (selected according to local tissue quality) were not recorded precisely at the time and cannot be reconstructed in retrospect. An additional count in peripheral retina of aardvark 2 has provided a new lower limit of 163,000 photoreceptors (Table S1, Abstract line 38, Results line 465, Discussion line 760).

2. (Lines 486-501)

The proportions of dual and single pigment cones are provided as ranges (e.g., between 35% and 96%). In some cases, the range is rather large. It would be helpful to include the actual data, regardless of the sample size, to know whether these variations are due to natural differences or a few outliers.

We thank the reviewer for this suggestion that has led us to have a second, more detailed look at our material. We have added the requested cone data for the various sampling regions in Table S2 and for one region in Fig. 8. The sampling regions are indicated in Fig. S1. For Fig. 8 we did additional counts that brought the insight that there actually is a clear cone density decrease from the streak to peripheral retina. The main text and discussion are modified accordingly (l. 509ff and 891-899).

3. (Figure 6D-E) The resolution of these images (at least in the PDF version available for review) is insufficient to evaluate the descriptions provided in the text. For example, it is unclear how the authors concluded that there is only one synaptic ribbon in a rod spherule. Similarly, it is difficult to verify whether there are few or no ribbon at the S cone pedicles.

We have replaced Fig. 6D1-3 (now Fig. 7D1-3) with an image at higher magnification to better show the ribbon localization relative to an S cone pedicle, and to better show the typical horseshoe shape of some rod-associated ribbons. We have left 7E1-3 unchanged, because in the digital publication, readers can zoom into the figures to see more detail.

Additionally, the images (Fig. 6E2, E3) show large, intensely stained CtBP2 blobs in addition to punctate stainings. Are these blobs associated with rod spherules? Furthermore, there are apparent holes in the OPL where no CtBP2 labeling is visible (Fig. 6E2). Are these areas devoid of photoreceptor terminals?

The stack in Fig. 6E (now Fig. 7E) is of 3 focal images spaced 0.5 µm apart. As the retinal piece was not perfectly flat, in some places the stack included the CtBP2-labelled INL somata (large green blobs, also seen in 7D), and it missed some ribbons located in the outer OPL (seemingly ribbon-free patches). This is now stated in the legend to Fig. 7. The fact that CtBP2 labels somata in the INL is also mentioned in the discussion (l. 916-917).

**Reviewer #2:**

Given the unique chance of investigating the eye of the aardvark *Orycteropus afer*, Peichl et al. present their observations on eye morphology and retinal features. The study is valuable since the authors have for the first time thoroughly documented features of the aardvark eye with modern techniques, laying the basis for broader comparative anatomical comparison. Overall, the paper is well written, presents unique research, and reports results compellingly. The main recommendation is making the link to ancestral relationships and morphological evolution more cohesive. Changes to some figures are recommended. Other minor adjustments are also suggested.

Thank you for this positive overall appraisal.

The authors set an expectation of discussing their findings from an evolutionary perspective by describing aardvarks as “living fossils” (63-65) and evaluating the reported divergence from the mammalian scheme of having both rods and cones (72-73). Although compelling, the evolutionary implications are somewhat fragmented, and would be strengthened by including:
- A phylogenetic tree or another schematic (either in the introduction or the discussion) to support comparisons across species and demonstrate the aardvark’s relation to other species.
- Brief comparison of aardvark retina to model species in the discussion.
- A dedicated discussion section summarising the evolutionary implications of the findings, elaborating on the notion that the aardvark does not seem to have a primordial retina. This would reinforce the final paragraph in the conclusions (855-862).

- We have added a phylogenetic tree of the Afrotheria as new Fig. 1 and modified the text accordingly.

- In the discussion, the comparison with the retinae of other species is extended by comparison of the aardvark cone properties to those of the few other studied Afrotheria. The evolutionary implication is that cone properties across Afrotheria may be as diverse as they are across mammals in general (l. 838 ff).

- We have decided against a separate dedicated section about the aardvark eye features from an evolutionary perspective, because we don’t have enough comparative data for a meaningful discussion. We think that the brief discussion about cone opsins in the Conclusion is sufficient to make the point that the aardvark retina is not primordial.

The methodology section is concise yet thorough, clearly outlining a systematic approach. Limitations are clearly identified throughout the methods sections. However:
- The authors do not justify why they chose to puncture behind the cornea (102) and why 4% formalin fixation for 24 h (103) was chosen. Some reasoning around this may alleviate concerns of over-fixation, for example if this protocol has been published elsewhere or has been previously tested on other tissue.

Puncturing of the eye, or cutting it halfway open, behind the cornea for better fixative access to the vitreous and retina is a standard procedure used by us and others (e.g., dog eyes: Peichl (1992) JCN 324: 590-602; seal eyes: Peichl & Moutairou (1998) EJN 10: 2586-2594; sheep eyes: Shinozaki et al. (2010) JCN 518: 2305–2315). Fixation with 4% formalin for 24h or even longer has been used when no other options were available (e.g., in field work settings) and has yielded satisfactory immunolabelling results (e.g., seal eyes: Peichl & Moutairou (1998) EJN 10: 2586-2594; rhinoceros eyes: Coimbra & Manger (2017) JCN 525: 2484–2498). As these protocols are pretty standard when handling ‘exotic’ eyes, we think it is not necessary to give references.

The results section is detailed and well written, with frequent reference to figures which were overall informative. Testing for hypothyroidism was a convincing way of assessing whether the aardvark retinae were aberrant. Checking heterochromatin distribution to confirm photoreceptor identities was a nice touch, and the related discussion of semi-inverted heterochromatin contextualised these results. Overall, the discussion also communicated well, and the included subsections lend themselves well to supporting the results. The figures are mostly good, but some changes are warranted:
- Figure 2: Add proper scale bars to images. These would be more informative than estimating from graph paper in Figure 2C.

We have retouched the graph paper background in Fig. 2B, C (now Fig. 3B, C) and introduced scale bars in A and C. We think D needs no scale bar, because its size can be judged from C.

- Figure 5 A, C, H, J: Would improve legibility to include the target protein in the confocal images (i.e., “DAPI” and “rho4D2”), as done for ‘NeuN’ and ‘Gln synthase’ in Figure 4. At least in the first images where used. Then, representing stains in the same colour consistently will help legibility (e.g. instead varying between blue, grayscale, and red for DAPI).

We have added the target proteins in the images, using RH1 for the mammalian rod opsin as suggested below. We have changed the colour of the RH1 label from green to yellow in Fig 5A-C (now Fig. 6A-C), because that gives a better contrast. For a consistent colour representation of the DAPI stain, we have changed the DAPI label to blue in images 5I, J (and correspondingly have changed the EC and HC labels to yellow for optimal contrast). However, converting images D-H from greyscale to blue would have reduced contrast too much, and the specific details of the different nuclear architectures would have been lost. So here we decided to stay with the greyscale images.

Actually, the S opsin labels in Fig. 6 (now Fig. 7) also are coloured inconsistently, some red, some magenta. For the benefit of red-green blind readers, we have used magenta when only two colours (magenta and green) were needed, but have used red when the blue channel was needed for the DAPI label. We think that is an acceptable compromise.

- Figure 5B: the figure legend states ‘Clearly, the vast majority of ONL somata belong to rods.’ Authors should demonstrate the association between rhodopsin and DAPI by showing a panel with the ONL zoomed in. Moreover, pointing out where DAPI was not associated with rhodopsin would further emphasise this.

In Fig. 5 (now Fig. 6), as new part 6C we have added a high-power image of the ONL showing an example of a RH1-negative cone soma surrounded by RH1-positive rod somata.

- Figure 6D: To better appreciate the localisation of ribbons and lack of co-localisation with S-opsin, an example image at higher magnification (or digital zoom) of the OPL would be beneficial.

We have replaced 6D1-3 (now Fig. 7D1-3) with an image at higher magnification to better show the ribbon localization relative to an S cone pedicle, and also the typical horseshoe shape of some rod-associated ribbons. The drawback of the higher magnification is that the field only contains one cone pedicle. We think that this is acceptable as Fig. 7E shows the lack of ribbon clusters in all cone pedicles present.

- Including some of the images used to determine photoreceptor densities (rods and cones) from different retinal regions would be instructive, for example by stitching together flat-mount sample fields (if contiguous), demonstrating periphery vs. central retina. Otherwise, a topographic schematic could summarise the same information.

This relates to the suggestions of reviewer 1. We consider it most instructive to provide these detailed data in the form of tables and graphs. Hence we have added Figures 8 and S1, and Tables S1 and S2, to the manuscript.

Other minor adjustments:

- 246: There is no curvature estimation in Figure 2A. Perhaps intention was to demonstrate that the cornea had collapsed? If so, reference for Figure 2A should appear after ‘[…] because the cornea had collapsed’, not after ‘[…] its original curvature was estimated’.

Yes; we have repositioned the image reference.

- 294: Figure 2A, D are more informative than Figure 1 here.

Yes; we have changed the figure reference to “(c.f. Fig. 3C, D)”.

- 279: The optic disk appears to be in a more nasal position on the temporal-nasal axis, as opposed to central.

The optic disc (OD) appears in in a nasal position in image 3D, because it shows an oblique view of the eyecup. We have now added a pointer to the OD position in image 3C, a frontal view of the eyecup. Because of a fold in the retina, the OD is not easily visible in 3C, but we have checked its position while isolating the retina and determined that the OD is located rather centrally on the temporal-nasal axis.

- 309: Add ‘(CC)’ after ‘choriocapillaris’, as CC is used as an abbreviation later in the text.

Done

- 331, 439, 522: The occasional deference to the discussion section is appropriate, although the consistent use of ‘(see Discussion)’ is preferred over statements like ‘This is addressed in the Discussion’.

We have changed that in most cases. In l. 451-452, we have kept the original wording as integral part of the sentence.

- 391: Rh1 is implied, but text only states ‘rod opsin’. Given existence of Rh2 (although not in mammals), it would reduce ambiguity to specify that the rhodopsin is Rh1.

We agree and have added RH1 here and in Table 1 and the labelling of Fig. 6; RH1 is what the antibody rho4D2 recognises.

- 519: The shape of the ribbon is not easily discernible from Figure 6E2. A higher zoom would help support the assertion.

The new Fig. 7D is at higher magnification and clearly shows the horseshoe shape of some ribbons. See response to reviewer 1 above.

- 532: For more precise language, change ‘open’ to ‘unknown’ in ‘[…] it remains open whether PNA […]’.

Done

- 596-598: The sentence about the repeated claim of aardvarks lacking cones can be excluded, as the point is made well-enough in the introduction.

Here we want to point out that Franz’s scientific error / misinterpretation has a long lifespan in standard zoological books and repositories. Hence it was worthwhile to revisit the issue. This point is not made in the Introduction, so we would like to keep this sentence.

- 621-623: The reference to a YouTube video is not needed, as Figure 1 is convincing.

Okay, this reference has been removed.

- 640: The idea of a mixed tapetum type is rather quickly dismissed and would benefit from justifying why the authors think a mix is unlikely. A brief comment would suffice.

We have added a short explanation and a ‘disclaimer’ on l. 706-709.

- 776: the authors should cite the specific works instead of ‘references therein’.

We have added a reference and think that these two references are sufficient to make the point (l. 869).

- 773-775: Would be easier to parse as ‘Aardvarks may use different sensitivities of pure L and S cones vs. dual pigment cones for colour vision’.

We have modified the sentence and hope that it reflects what the reviewer intended (l. 867-868).

Overall, a very nicely presented assessment of the aardvark eye structure and photoreceptor identities. Looking forward to seeing the final publication, as well as the follow-up on inner-retina cell types.

We are working on the follow-up. However, we have removed the reference to it (l. 84/85) to not put us under time pressure for its publication.
